# Sphingosine-1-phosphate modulates PAR1-mediated human platelet activation in a concentration-dependent biphasic manner

**DOI:** 10.1038/s41598-021-94052-4

**Published:** 2021-07-28

**Authors:** Haonan Liu, Molly L. Jackson, Lucy J. Goudswaard, Samantha F. Moore, James L. Hutchinson, Ingeborg Hers

**Affiliations:** 1 School of Physiology, Pharmacology and Neuroscience, Biomedical Sciences Building, University of Bristol, Bristol, BS8 1TD UK; 2 Population Health Sciences, Oakfield House, University of Bristol, Bristol, BS8 2BN UK

**Keywords:** Cell biology, Pharmacology

## Abstract

Sphingosine 1-phosphate (S1P) is a bioactive signalling sphingolipid that is increased in diseases such as obesity and diabetes. S1P can modulate platelet function, however the direction of effect and S1P receptors (S1PRs) involved are controversial. Here we describe the role of S1P in regulating human platelet function and identify the receptor subtypes responsible for S1P priming. Human platelets were treated with protease-activated receptor 1 (PAR-1)-activating peptide in the presence or absence of S1P, S1PR agonists or antagonists, and sphingosine kinases inhibitors. S1P alone did not induce platelet aggregation but at low concentrations S1P enhanced PAR1-mediated platelet responses, whereas PAR1 responses were inhibited by high concentrations of S1P. This biphasic effect was mimicked by pan-S1PR agonists. Specific agonists revealed that S1PR_1_ receptor activation has a positive priming effect, S1PR_2_ and S1PR_3_ have no effect on platelet function, whereas S1PR_4_ and S1PR_5_ receptor activation have an inhibitory effect on PAR-1 mediated platelet function. Although platelets express both sphingosine kinase 1/2, enzymes which phosphorylate sphingosine to produce S1P, only dual and SphK2 inhibition reduced platelet function. These results support a role for SphK2-mediated S1P generation in concentration-dependent positive and negative priming of platelet function, through S1PR1 and S1PR4/5 receptors, respectively.

## Introduction

Platelet primers are bioactive molecules that do not induce platelet activation by themselves but modulate platelet responses to physiological agonists such as thrombin and adenosine diphosphate (ADP)^1^. Diseases such as chronic inflammation, type II diabetes and cardiovascular disease feature elevated circulating concentrations of certain growth factors, glycoprotein hormones and cytokines, alongside enhanced receptor expression levels^2^. There is evidence that circulating sphingosine-1-phosphate (S1P) is elevated in obesity, atherosclerosis and cancer^3,4^, whereas S1P is reduced upon ischemic stroke^5^ and myocardial infarction^6^.

Intracellular S1P concentrations are kept low by sphingosine-1-phosphate phosphatase (SPP) and sphingosine-1-phosphate lyase (SPL) enzymes. Platelets do not express SPP and SPL and exhibit constitutive high sphingosine kinase (SphK) activity^7^. Hence, platelets can produce and store a large amount of S1P^8^. Previous studies have demonstrated that thrombin and protease-activated receptor-1 (PAR1) mediated platelet activation results in S1P synthesis and release from platelets, which was at least partially dependent on thromboxane A_2_ (TxA_2_) formation and activation of the thromboxane (TP) receptor^9,10^. Furthermore, S1P synergizes with thrombin to promote tissue factor expression on endothelial cells, propagating a positive feedback loop^11^.

However, there is controversy in the literature about mechanisms of S1P synthesis and release by platelets and how endogenous S1P affects platelet function. For example, Münzer et al.^12^ demonstrated that SphK1 negatively regulates mouse platelet function and SphK1 knock out significantly promoted platelet function. In contrast, the study by Urtz et al.^13^ showed that SphK2 is the major isoform of SphK in mouse platelets and responsible for platelet S1P synthesis, with deletion of SphK2 greatly reducing murine platelet aggregation. This is concurrent with mouse platelet transcriptome data, where SphK1 transcript level is much lower than SphK2^7^. Although mouse studies indicate a potential negative and positive role for SphK1 and SphK2, respectively^12,13^, the contribution of sphingosine kinases to human platelet function is still largely unknown. Transcriptome data indicate that, in contrast to murine platelets, human platelet SphK1 (RPKM of 24.7) is expressed at a much higher level than SphK2 (RPKM of 0.5)^7^, however whether this is reflected in a more significant role in human platelets remains to be confirmed.

Multiple studies have reported that platelets release S1P upon platelet activation by various agonists^8,14–17^, and studies by the Whiteheart group indicate that this is through release from the α-granules^14^. Furthermore, it was reported that human platelets express S1P receptors and platelet-synthesized S1P might therefore signal in both autocrine and paracrine manner^18^. Indeed, several studies indicate that exogenous S1P can directly activate human platelets causing shape change and aggregation^18–21^. However, other studies reported no direct effect of S1P on platelet activation, but instead found that S1P can modulate platelet function^22–24^. Both enhancing as well as inhibitory effects of S1P have been reported with roles suggested of S1PR1, S1PR2 and S1PR4^13,17,22–25^. However, the use of different species, agonists and often supraphysiological concentrations of S1P (> 10 μM), make it difficult to draw conclusions on the physiological implications of these findings in human.

The aim of this study was therefore to investigate the effect of various concentrations of S1P on human platelets and determine the underlying receptors by which it may modulate human platelet function. Here we show that exogenous S1P concentration-dependently alters platelet function, with low concentrations of S1P priming platelet function and high concentrations of S1P inhibiting platelet responses. Our data furthermore support a role for S1PR_1_, S1PR_4_ and S1PR_5_ receptor subtypes in modulating human platelet function.

## Results

### Sphingosine-1-phosphate modulates PAR1-AP mediated aggregation in a concentration-dependent, biphasic manner

**Figure 1:**
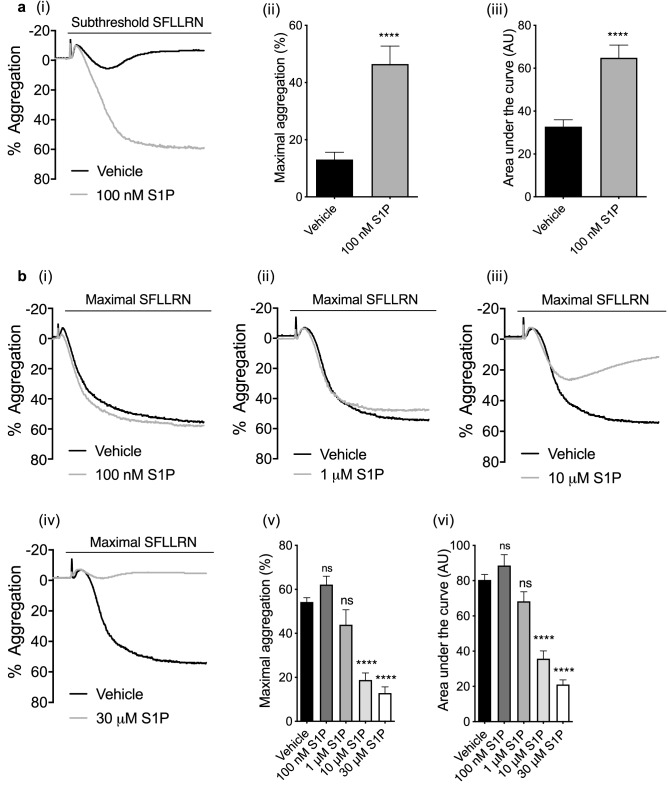
S1P modulates PAR1-AP induced platelet aggregation in a concentration dependent biphasic manner. Washed human platelets (2 × 10^8^/mL) were pre-incubated with vehicle (0.2% methanol), 100 nM S1P and high concentrations of S1P for 5 min. Platelet aggregation was induced by subthreshold (0.6–1.0 μM) or maximal (1.5–2.0 μM) concentrations of PAR1-AP and recorded for 5 min. (**a**) (i) Representative traces of subthreshold and maximal aggregation induced by PAR1-AP in the absence or presence of 100 nM S1P. (ii) Bar graph of quantified percentage maximum aggregation induced by PAR1-AP alone or with the presence of 100 nM S1P. (iii) Bar graph of AUCs induced by PAR1-AP alone or with the presence of 100 nM S1P. AU: arbitrary unit. Data are plotted as mean ± standard error of the mean. N = 17 Statistical analysis: Student’s paired t-test. NS, no statistical significance. ****P < 0.0001 (**b**) (i–iv) Representative traces of aggregation induced by maximal concentration of PAR1-AP in the absence or presence of 100 nM, 1 μM, 10 μM and 30 μM S1P. (v) Bar graph of quantified percentage maximum aggregation induced by high PAR1-AP alone or in the presence of various concentrations of S1P (100 nM, 1 μM, 10 μM, 30 μM). (vi) Bar graph of AUCs induced by high concentration PAR1-AP alone or in the presence of various concentrations of S1P (100 nM, 1 μM, 10 μM, 30 μM). Data are plotted as mean ± standard error of the mean. n = 12. Statistical analysis: Student’s paired t-test. NS, no statistical significance; ****P < 0.0001.

S1P alone was unable to induce platelet aggregation, shape change or Ca^2+^ mobilisation at a range of concentrations (100 nM–10 μM, not shown). Aggregation responses to subthreshold concentrations of the PAR1 peptide SFLLRN were however significantly increased in the presence of 100 nM S1P (Fig. 1ai), with an increase in peak aggregation (Fig. 1aii) and the area under the curve (Fig. 1aiii). In contrast, maximal platelet responses to 1.5–2 μM SFLLRN were unaltered by 100 nM S1P (Fig. 1bi, v, vi). Interestingly, 1 μM S1P did not have any effect on PAR-mediated platelet aggregation (Fig. 1bii, v, vi), but increasing the S1P concentration to 10 μM and 30 μM strongly reduced PAR-mediated platelet aggregation (Fig. 1biii, iv), resulting in a significant decrease in peak aggregation and AUC (Fig. 1bv, vi). These findings indicate that exogenous S1P can modulate PAR-mediated platelet activation in a concentration-dependent, biphasic manner. S1P was also able to modulate the aggregatory response to CRP: 100 nM S1P was able to potentiate the aggregation induced by a subthreshold concentration of CRP (Supp Fig. 1a), while 10 μM S1P reduced CRP-induced platelet aggregation (Supp Fig. 1b).

### Biphasic effect of S1P on integrin activation and α-granule secretion

**Figure 2:**
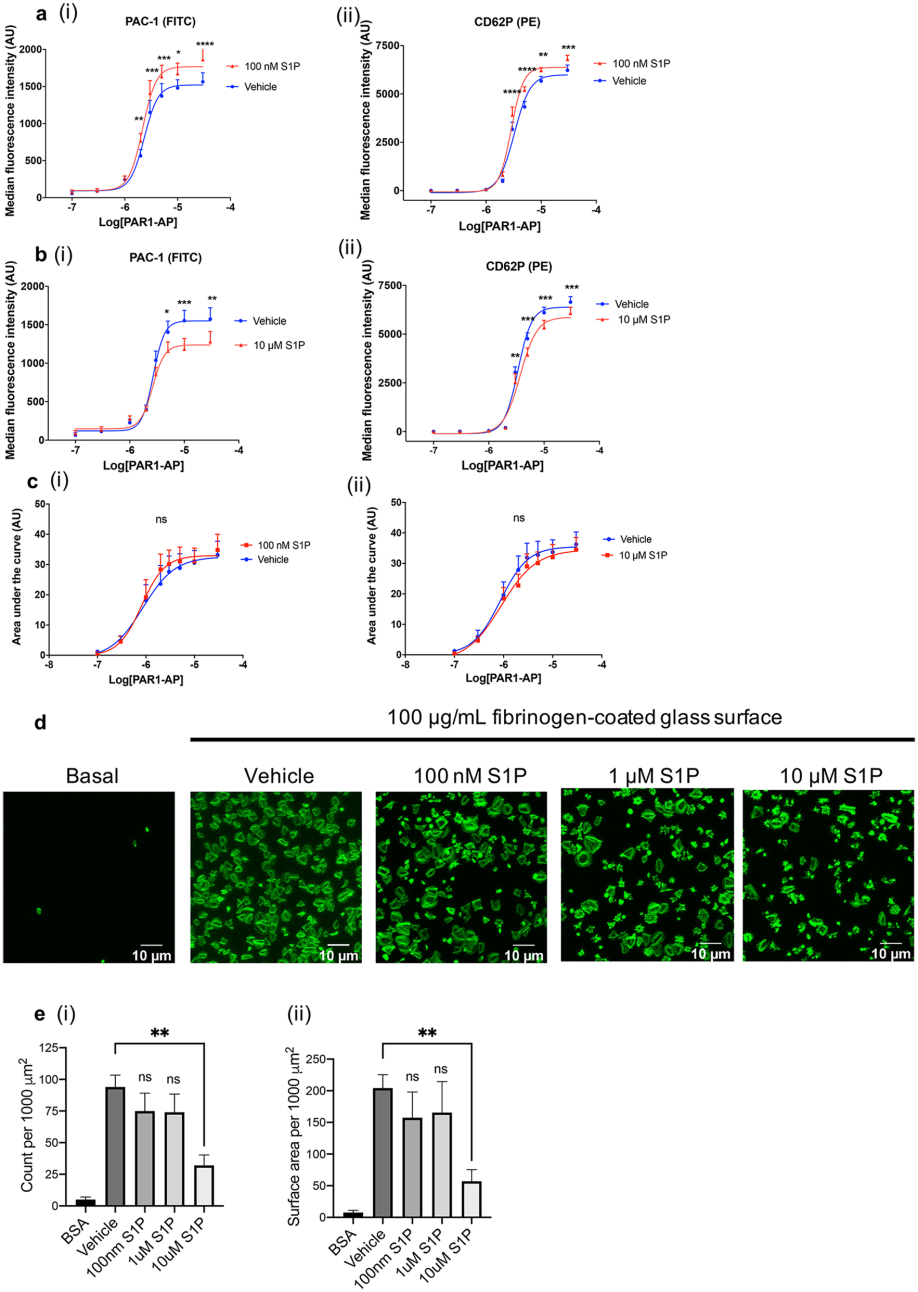
Biphasic effect of S1P on integrin α_IIb_β_3_ activation, P-selectin expression and platelet spreading on fibrinogen coated surface. (**a**, **b**) Washed platelets (2 × 10^7^/mL) were incubated with vehicle (0.2% methanol) or S1P (100 nM, 10 μM, 10 min), before stimulation with increasing concentrations of PAR1-AP for 10 min. PAC-1 was used to measure α_IIb_β_3_ integrin activation (ai, bi) and CD62P was used to measure P-selectin expression (aii, bii). Results are expressed as median fluorescent intensity. AU: arbitrary unit. N = 10. Statistical analysis was by matched two-way ANOVA with Bonferroni post hoc multiple comparisons to assess significance for effect of S1P at each concentration of PAR1-AP as indicated. * *P* < 0.05, **P < 0.01, ***P < 0.001, ****P < 0.0001. (**c**) Washed human platelets (2 × 10^8^/mL) loaded with Fura-2 were preincubated with vehicle, (i) 100 nM S1P or (ii) 10 μM S1P before stimulation with increasing concentrations of PAR1-AP. Area under the curve is derived from the ratio of fluorescence at the wavelengths 340:380 nm. Statistical analysis was by matched two-way ANOVA with Bonferroni post hoc multiple comparisons to assess significance for effect of S1P at each concentration of PAR1-AP as indicated, ns = non-significant. (**d**) Washed platelets (2 × 10^7^/mL) were incubated with vehicle (0.2% methanol) or various concentrations of S1P (100 nM, 1 μM, 10 μM) for 10 min. Platelets were then left to adhere onto a 13 mm glass coverslip coated with 100 μg/mL fibrinogen (60 min, 30 °C). Samples were fixed (4% PFA) before permeabilization (0.1% triton) and staining with ActinGreen 488 ReadyProbes reagent. (**e**, i) Quantification of data shown in (**d**) expressed as a bar graph of the total number of platelets adhered. (**e**, ii) Quantification of data shown in (**d**) expressed as a bar graph displaying the average platelet area (μm^2^) (N = 5). Three images were taken per well. Average cell area was measured per 1000 μM^2^, data are plotted as mean ± SEM. N = 5. Statistical analysis: One-way ANOVA with Dunnett’s test. **P < 0.01.

Integrin α_IIb_β_3_ inside-out signalling leads to a change in conformation of the integrin that allows binding of fibrinogen and subsequent platelet aggregation. To study whether S1P affects integrin α_IIb_β_3_ inside-out signalling, we evaluated binding of PAC1, an antibody which recognises the activated conformation of integrin α_IIb_β_3_. We observed enhanced PAR-1-mediated PAC1 binding in the presence of 100 nM S1P (Fig. 2ai), whereas PAC1 binding in the presence of 10 μM S1P was reduced (Fig. 2bi). Similar results were found when studying PAR1-mediated expression of the α-granule marker P-selectin, although the extent of the effect was smaller (Fig. 2aii, bii). One of the potential mechanisms by which S1P affects platelet function is by modulating [Ca^2+^] mobilisation, an important signalling event involved in integrin regulation, granule secretion and aggregation. However, neither 100 nM or 10 μM S1P exhibited a significant effect on Ca^2+^ mobilisation induced by increasing concentrations of PAR1-AP (Fig. 2ci, ii).

### The effect of S1P on platelet adhesion and spreading

Integrin α_IIb_β_3_ outside-in signalling consolidates platelet aggregation and thrombus formation^26^. Here we studied integrin α_IIb_β_3_ outside-in signalling by allowing platelets to adhere and spread on a fibrinogen-coated surface. We found that 100 nM S1P had no effect on the number of adhered platelets and average platelet surface area (Fig. 2d, 2ei, ii). However, increasing the S1P concentration to 10 μM blocked fibrinogen-mediated adhesion and spreading (Fig. 2d, 2ei, ii). S1P at a concentration of 10 μM increased the percentage of platelets in phase I and phase II (compact shape and filopodial extension, respectively) while reducing the number of platelets in phase IV (fully spread, Supp Fig. 2a).

### Pan-S1P receptor agonists also exhibit concentration-dependent biphasic modulation of PAR-induced platelet aggregation

**Figure 3:**
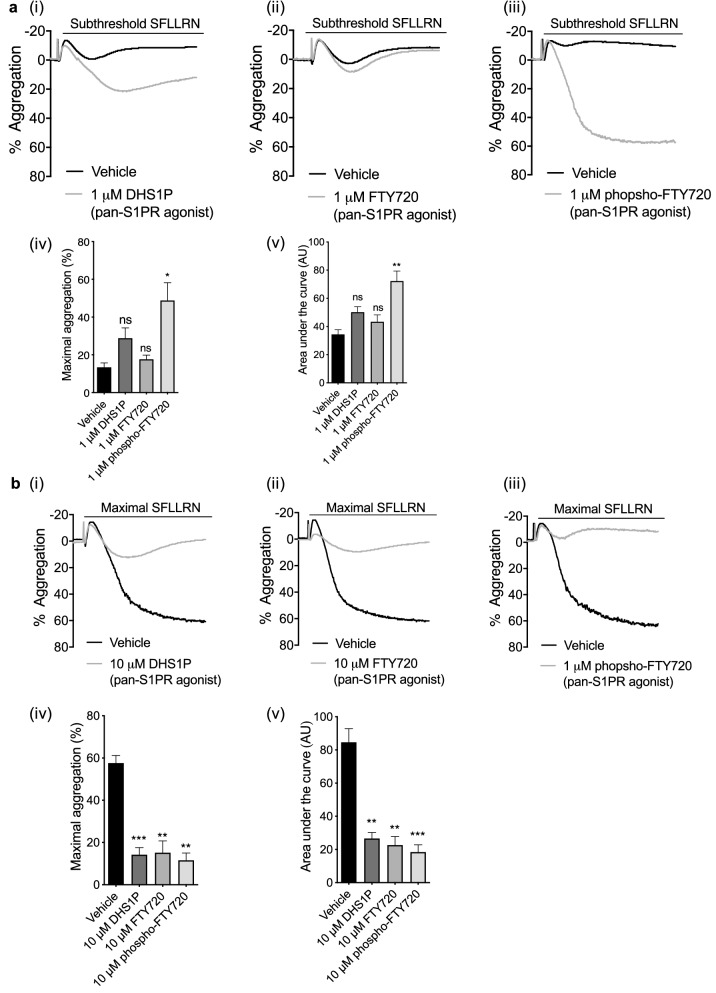
Pan-S1PR agonists modulate PAR-induced platelet aggregation. Washed human platelets (2 × 10^8^/mL) were treated with vehicle control or S1PR agonist for 5 min. Platelet aggregation was induced by a subthreshold (0.6–1.0 μM) concentration of PAR1-AP and recorded for 5 min. (**a**) (i–iii) Representative traces of PAR1-AP induced platelet aggregation in the absence or presence of 1.0 μM DHS1P, 1.0 μM FTY720 or 1.0 μM FTY720 phosphate. (iv) Bar graph of quantified percentage maximum aggregation induced by PAR1-AP alone or in presence of pan-S1PR agonists. (v) Bar graph of AUCs induced by PAR1-AP alone or in the presence of S1PRs pan agonists. AU: arbitrary unit. Data are plotted as mean ± standard error of the mean. N = 6. Statistical analysis: Student’s paired t-test. NS: no statistical significance; *P < 0.05, **P < 0.01. (**b**) (i–iii) Representative traces of PAR1-AP induced platelet aggregation in the absence or presence of 10 μM DHS1P, 10 μM FTY720 or 10 μM FTY720 phosphate. (iv) Bar graph of quantified percentage maximum aggregation induced by PAR1-AP alone or in presence of pan-S1PR agonists. (v) Bar graph of AUCs induced by PAR1-AP alone or in the presence of S1PRs pan agonists. AU: arbitrary unit. Data are plotted as mean ± standard error of the mean. N = 6. Statistical analysis: Student’s paired t-test. NS: no statistical significance; *P < 0.05, **P < 0.01, ***P < 0.001.

The pan-S1PR agonists dihydro-S1P (DHS1P)^27^, FTY720 and phospho-FTY720^28^ at 1 μM all enhanced maximal aggregation stimulated by subthreshold PAR1-AP and AUC (Fig. 3ai, ii, iii), although this only reached significance for phospho-FTY720 (Fig. 3aiv, v). Conversely, increasing the pan-S1PR agonists concentrations to 10uM resulted in strong inhibition of maximal platelet responses to PAR1-AP (Fig. 3bi, ii, iii, iv, v). Thus, as with S1P, pan-S1P receptor agonists modulation of PAR1-mediated platelet aggregation is biphasic. These data demonstrate that the dual effect of S1P is likely to be mediated via activation of S1P receptors.

### Platelet aggregation is enhanced by S1PR_1_ activation, but reduced by S1PR_4/5_ activation

**Figure 4:**
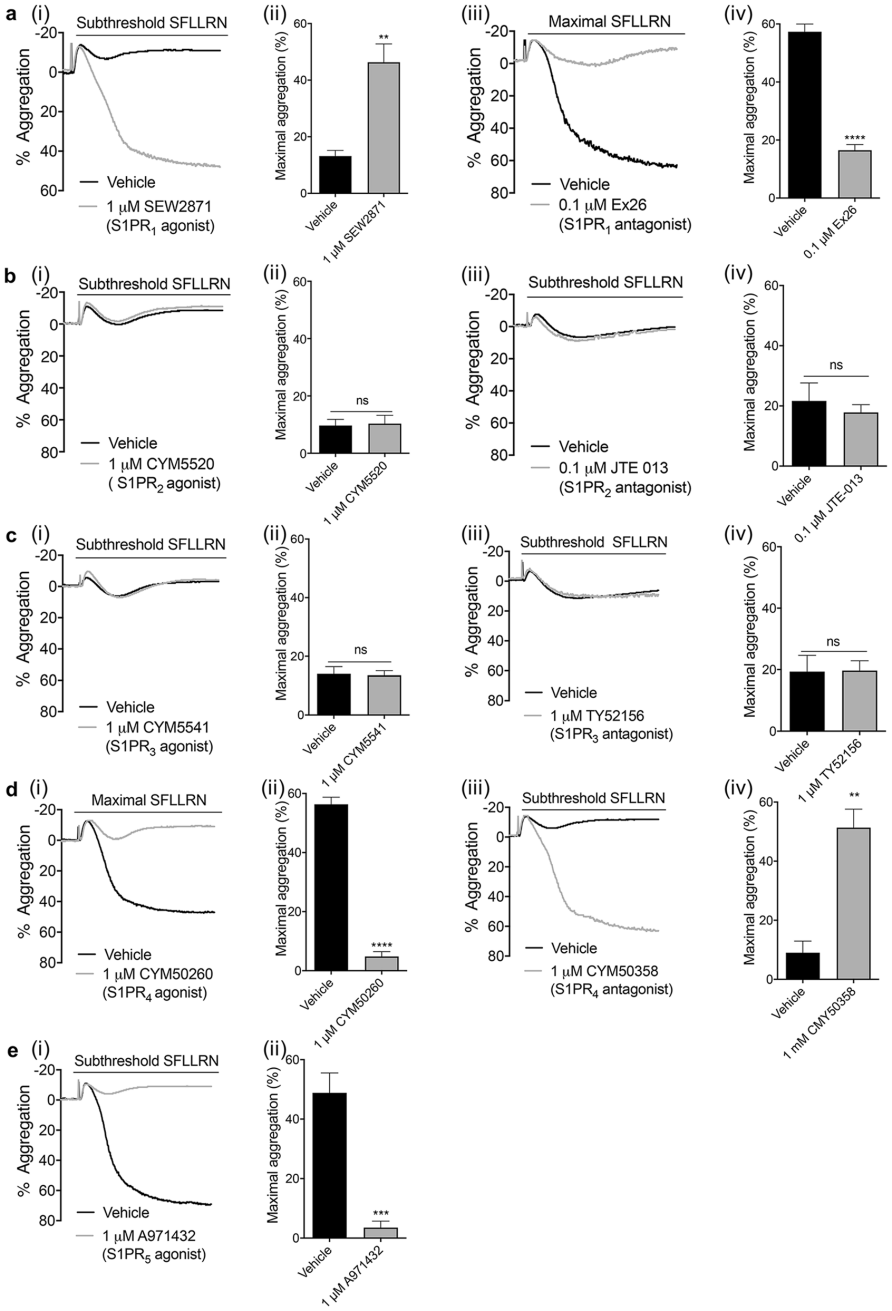
Effect of S1P receptor agonists and antagonists on PAR-mediated aggregation. Washed human platelets (2 × 10^8^/mL) were treated with vehicle (0.2% methanol) or S1PR agonists for 5 min. Platelet aggregation were induced by subthreshold (0.6–1.0 μM) or maximal (1.5–2.0 μM) concentrations of PAR1-AP and recorded for 5 min. (**a**–**e**) (i) Representative traces of platelet aggregation induced by subthreshold PAR1-AP in the absence or presence of the (**a**) S1PR_1_ agonist SEW2871, (**b**) S1PR_2_ agonist CYM5520, (**c**) S1PR_3_ agonist CYM5541, (**d**) S1PR_4_ agonist CYM50260 or (**e**) S1PR_5_ agonist A971432. (ii) Bar graph of quantified percentage maximum aggregation induced by PAR1-AP alone or in presence of S1P receptor agonists. AU: arbitrary unit. Data are plotted as mean ± standard error of the mean. Statistical analysis: Student’s paired t-test. NS: no statistical significance; **P < 0.01, ***P < 0.001, ****P < 0.0001. (**a**–**d**) (iii) Representative traces of PAR1-AP induced platelet aggregation in the absence or presence of (**a**) S1PR_1_ antagonist Ex26, (**b**) S1PR_2_ antagonist JTE-013, (**c**) S1PR_3_ antagonist TY52156 and (**d**) S1PR_4_ antagonist CYM50358 hydrochloride. (iv) Bar graph of quantified percentage maximum aggregation induced by PAR1-AP alone or in presence of S1P receptor antagonists. AU: arbitrary unit. Data are plotted as mean ± standard error of the mean. Statistical analysis: Student’s paired t-test. NS: no statistical significance; **P < 0.01, ****P < 0.0001.

We next investigated which S1P receptor subtypes are involved in S1P-mediated modulation of platelet function by incubating platelets with S1PR selective agonists and antagonists. The S1PR_1_ agonist SEW2871^29^ significantly increased PAR1-mediated platelet aggregation (Fig. 4ai, ii), whereas the S1PR_1_ antagonist Ex26^30^ decreased high PAR1-AP mediated platelet function (Fig. 4aiii, iv), supporting a role for S1PR_1_ in enhancing platelet function. The respective S1PR_2_ and S1PR_3_ allosteric agonists CYM5520 and CYM5541^31,32^ and respective antagonists JTE-013^33^ and TY52156^34^ had no effect on PAR1-mediated platelet aggregation, indicating these receptors are not involved in the platelet S1P response (Fig. 4b, c). In contrast, activation of S1PR_4_ or S1PR_5_ receptors, with CYM50260^24^ and A971432^35^ respectively, caused strong inhibition of high PAR1-AP mediated platelet aggregation (Fig. 4di, dii, e). Conversely, antagonising S1PR_4_ with CYM50358 hydrochloride ^36^ significantly enhanced subthreshold PAR1-mediated platelet aggregation (Fig. 4diii, div). Commercial S1PR_5_ antagonists were not available to examine the effect of inhibiting S1PR_5_ on platelet function. Together, our data suggest that S1PR_1_ is responsible for the positive priming effect, whereas S1PR_4_ and S1PR_5_ mediate the inhibitory effects of S1P.

### S1PR_4_ and S1PR_5_ agonists and sphingosine kinase inhibitors reduce integrin α_IIb_β_3_ mediated platelet adhesion and spreading

**Figure 5:**
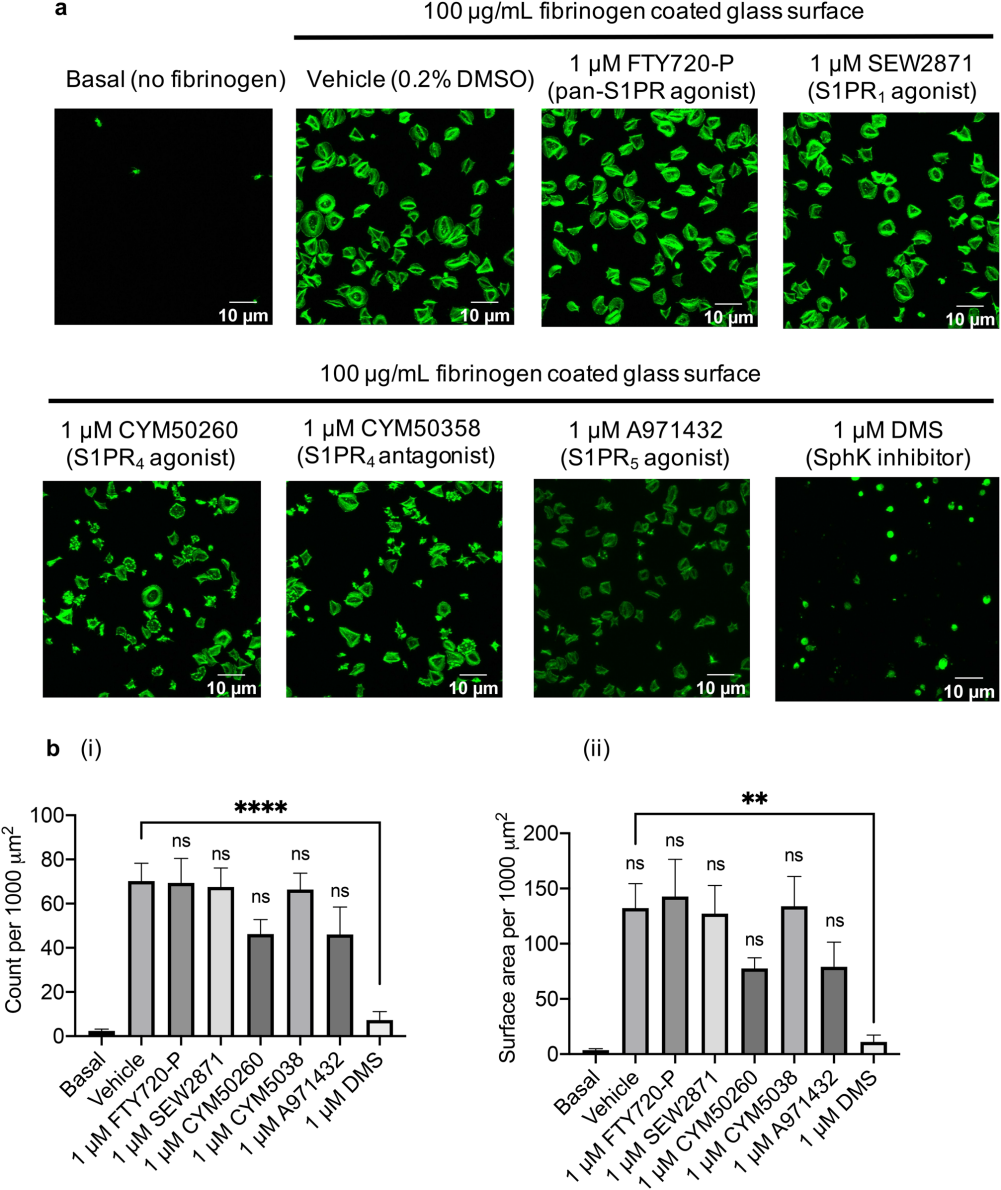
Platelet spreading on fibrinogen is inhibited by S1PR_4_ and S1PR_5_ agonists and SPHK inhibitor. Washed platelets (2 × 10^7^/mL) were incubated with vehicle control (0.2% methanol), S1P receptor subtype specific agonists or SphK inhibitor DMS for 10 min. Platelets were then left to adhere onto a 13 mm glass coverslip coated with 100 μg/mL fibrinogen (60 min, 30 °C). Samples were fixed (4% PFA) before permeabilization (0.1% triton) and staining with ActinGreen 488 ReadyProbes reagent. (**a**) Representative images of basal (no fibrinogen), vehicle control, 1 μM FTY720 phosphate, 1 μM SEW2871,1 μM CYM50260, 1 μM CYM50358 hydrochloride, 1 μM A971432, 1 μM DMS. (**b**) Bar graphs of the (i) total number of platelets and (ii) average platelet cell area of platelets adhered in the absence and presence of S1P receptor subtype specific agonists or SphK inhibitor. N = 5. Three images were taken from each well. Average cell area was measured per 1000 μm^2^, data are plotted as mean ± SEM. Statistical analysis: One-way ANOVA with Dunnett’s test. ns = not significant, **P < 0.01, ****P < 0.0001.

To investigate which S1P receptor subtypes inhibits platelet spreading, platelets were treated with S1PR agonists, antagonists or the non-selective sphingosine kinase inhibitor. Consistent with 100 nM S1P in earlier experiments, the pan-S1PR agonist FTY720-P at 1 µM had no effect on adhesion and spreading, as the number of adhered platelets and average platelet surface area remained unchanged (Fig. 5a and b). The S1PR_1_ agonist SEW2871 and S1PR_4_ antagonist CYM50358 hydrochloride also had no effect on platelet spreading, as was the case with S1PR_4_ agonist CYM50260 and S1PR_5_ agonist A971432. These compounds did also not affect the different phases of platelet spreading, apart from an increase in phase I (compact shape platelets) observed with CYM50260 compared to vehicle control (Supp Fig. 2b). In contrast, the SphK inhibitor DMS^37^ significantly reduced the number of adhered platelets and the average platelet surface area (Fig. 5a and b). This change is reflected in the phases of platelet spreading with DMS increasing the number of platelets in phase I (compact shape) and reducing the number of platelets in phase IV (fully spread) (Supp Fig. 2b).

### Sphingosine kinases regulate PAR1-mediated platelet aggregation

**Figure 6:**
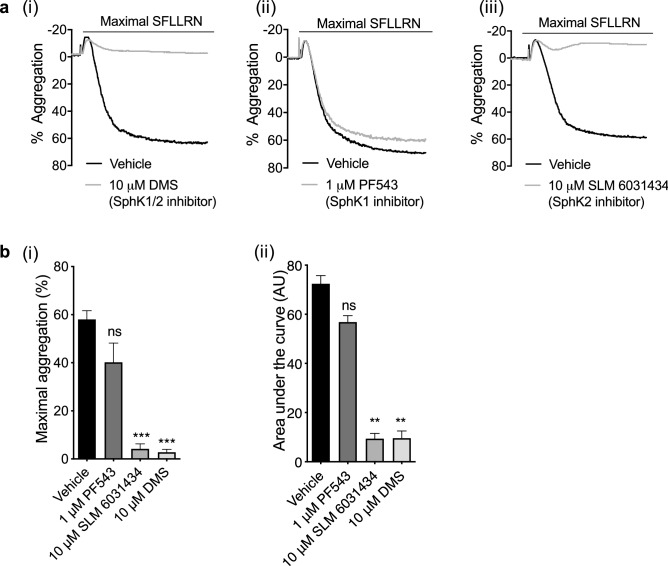
Inhibiting sphingosine kinases reduces PAR1-AP induced platelet aggregation. Washed human platelets (2 × 10^8^/mL) were pre-incubated with vehicle control (0.2% methanol) or sphingosine kinase inhibitors for 5 min. Platelet aggregation was induced by maximal (1.5–2.0 μM) concentration of PAR1-AP and recorded for 5 min. (**a**) Representative traces of maximal concentration PAR1-AP induced platelet aggregation in the absence or presence of (i) 10 μM SPHK1/2 inhibitor DMS (N, N-Dimethylsphingosine), (ii) 1 μM SPHK1 inhibitor PF543 hydrochloride or (iii) 10 μM SPHK2 inhibitor SLM 6,031,434, respectively. (**b**) Bar graphs of (i) quantified percentage aggregation and (ii) AUCs induced by maximal concentration of PAR1-AP alone or in the presence of SPHK inhibitors. AU: arbitrary unit. Data are plotted as mean ± standard error of the mean. N = 5. Statistical analysis: Student’s paired t-test. ns = not significant, *P < 0.05, **P < 0.01, ***P < 0.001.

Endogenous S1P is generated from sphingosine by the action of SphKs 1 and 2^38^. We incubated platelets with various SphK inhibitors to evaluate the contribution of sphingosine kinases to platelet function. PAR1-AP mediated aggregation and AUC was significantly reduced in the presence of the non-selective SphK1/2 inhibitor DMS^37^ (Fig. 6ai, bi, bii). The SphK1 inhibitor PF543 hydrochloride^39^ had no significant effect on aggregation (Fig. 6 aii, bi, bii), whereas strong inhibition was observed in the presence of the SphK2 inhibitor SLM 6031434^40^ (Fig. 6 aiii, bi, ii). The latter agrees with Urtz et al.’s observations^13^ in mouse platelets and indicate that SphK2 may also be the major sphingosine kinase isotype controlling S1P synthesis in human platelets. Indeed, our data demonstrate that sphingosine kinases play a role in aggregation of human platelets, possibly via de novo S1P synthesis and subsequent autocrine activation on the S1PR_1_ receptor.

## Discussion

**Figure 7:**
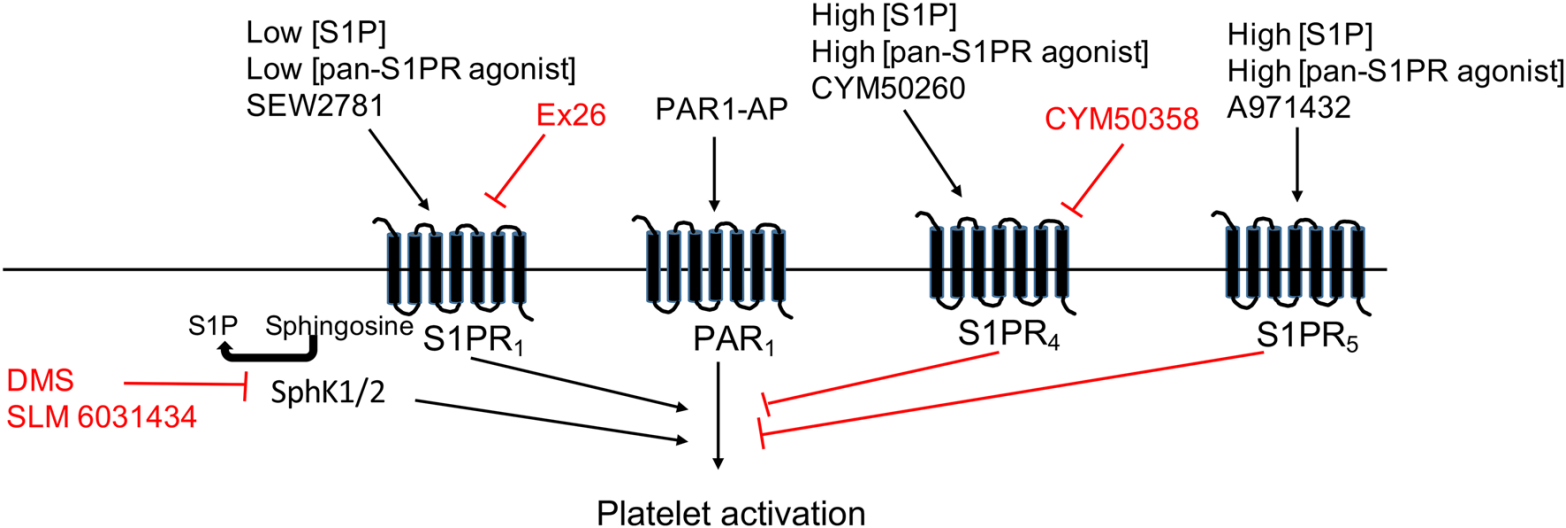
Summary of the biphasic modulation of platelets by S1P. Black arrows indicate activation, with red lines indicating inhibition.

This study demonstrates for the first time that exogenous S1P can concentration-dependently alter platelet function, with low concentrations of S1P priming platelet function and high concentrations of S1P inhibiting platelet responses. Our data indicate that the receptors involved are S1PR_1_, mediating the enhancing effect of S1P, whereas S1PR_4_ and S1PR_5_ are involved in S1P mediated inhibition of platelet function (Fig. 7). Furthermore, we found that both receptor antagonism by high S1P concentrations and inhibition of sphingosine kinases reduced PAR1-AP mediated platelet aggregation and platelet spreading, suggesting endogenous de novo synthesized S1P plays an important role in controlling platelet activation and change in morphology.

S1P exerted a characteristic, concentration-dependent biphasic effect on PAR-mediated platelet function as determined through platelet aggregation experiments. This modulatory effect of S1P is not agonist specific, as aggregation experiments using CRP also suggested that S1P can modulate the glycoprotein VI (GPVI) receptor signalling pathway. Integrin α_IIb_β_3_ activation and P-selectin exposure in response to PAR1-AP were enhanced in the presence of 100 nM S1P but inhibited in the presence of super-micromolar S1P. This is the first time the S1P biphasic effect has been observed in a platelet study. However, similar concentration-dependent biphasic effects of S1P have previously been observed in other cell types, including on ovarian cancer cell growth^41^, osteoblastic cell growth, T cell migration^42^ and calcium release in vascular smooth muscle cells^43^. The biphasic effect is likely to be due to differential signalling via S1P receptor subtypes, with the turning-point concentration dependent on the incubation time and/or cellular response measured. In our study, we also observed similar biphasic effects in platelets pre-incubated with the pan-S1PR agonists DHS1P, FTY720 and phosphorylated FTY720. However, DHS1P (1 μM) induced a milder enhancement in platelet aggregation compared to S1P, which may be due to DHS1P having a lower binding affinity for the S1PRs and/or that it cannot evoke intracellular effects such as calcium mobilization. Further increasing the DHS1P concentration resulted in platelet inhibition.

Of the S1PR receptor subtypes, S1PR_1_, S1PR_2_ and S1PR_3_ are widely expressed, while S1PR_4_ is predominantly expressed in lymphoid tissue and S1PR_5_ expressed in spleen and the central nervous system^44–46^. Previously it was suggested S1PR_1_, S1PR_2_, S1PR_4_ and S1PR_5_ are expressed in human platelets^20,24^. In this study, we probed S1P receptors on human platelets using commercially available pharmacological compounds. We found S1PR_1_ agonists enhanced platelet activation and the S1PR_1_ antagonist inhibited platelet activation and it is therefore likely that the positive priming effect of S1P is mediated through the S1PR_1_ receptor. S1PR_1_ is a G_αi/o _coupled receptor and the mechanism of potentiation of platelet function may therefore be similar to the G_αi/o _coupled ADP receptor P2Y_12_. Interestingly, no effect on platelet aggregation was observed with selective S1PR_2_ and S1PR_3_ agonists and antagonists, which suggests that S1PR_2_ and S1PR_3_ receptors either do not modulate platelet function or are not expressed on human platelets. The latter is in line with data published by Rowley et al.^7^ which did not find S1PR_2_ or S1PR_3_ transcriptome in human platelets. Of note is that proteomic studies have thus far failed to identify S1PR subtypes in platelets, likely to be due to it being more challenging to identify membrane protein/receptors. In addition, calcium mobilization assays demonstrated that S1P alone or in combination with PAR1-AP does not modulate platelet calcium signalling. This data further suggests that S1P is not activating S1PR_2_ or S1PR_3_, as these receptors have been reported to couple to G_αq_^47,48^. However, as the S1PR_2_ and S1PR_3_ agonists bind to different sites on the receptors than the orthosteric ligand S1P^31,32^, we cannot rule out that affinity and receptor binding sites differences might result in signalling differences or functional biases.

In contrast to S1PR_1_ agonists, pre-treatment of platelets with the S1PR_4_ agonist CYM50260 significantly inhibited PAR1-AP mediated aggregation. This is consistent with the study by Onuma et al.^24^ which demonstrated that high concentrations of S1P or S1PR_4_ agonist strongly inhibited collagen-mediated platelet function. Furthermore, our aggregation data indicate that antagonising S1PR_4_ significantly enhanced platelet aggregation. Together, these findings indicate that functional S1PR_4_ receptors are expressed on human platelets and play a role in negative regulation of platelet function by multiple agonists. Moreover, our aggregation data demonstrated for the first time that PAR1-mediated platelet activity was strongly reduced in the presence of S1PR_5_ agonist A971432. The mechanism by which S1PR_4_ and S1PR_5_ inhibit platelet function is presently unclear, as they reportedly couple to G_αi_ and/or G_α12/13_ in other cells types; pathways which in platelets are generally involved in increasing platelet function^49^. Although the S1PR_4_ agonist CYM50260 increased the number of platelets in a compact formation compared to vehicle, therefore demonstrating the inhibitory effect, the S1PR_4_ and S1PR_5_ agonists did not significantly alter the number of platelets adhered or surface area with adhered platelets. This suggests that the combination of platelet agonists and activation of S1PR_4_ and S1PR_5_ is important for the inhibitory effects. Several studies have indicated that S1PR_4_ and S1PR_5_ primarily couple to G_α12/13_ over G_αi/o_^50^ and in platelets, G_α12/13_ has been implicated in (i) negative regulation of G_q_-mediated signalling^51^ and (ii) both positive and negative regulation of Rho family small GTPases^51,52^. Temporal regulation of signalling by G_α12/13_ may therefore provide a potential explanation for the negative priming effect of S1PR_4_ and S1PR_5_ on platelet function.

Previous studies showed that impaired platelet S1P synthesis in SphK2 deficient mice protected mice from in vivo arterial thrombosis, without associated bleeding, indicating a role for S1P in thrombosis, likely through an effect on S1PR1^13^. However, other studies suggest that S1PR1 is not expressed in murine platelets and that S1P has little effect on murine platelet function^53^. Our findings of biphasic effects of S1P on human platelet function raises the intriguing question of how this translates into a physiological role in platelet aggregation and thrombus formation in humans. Based on our data, we propose that S1P released by platelets initially promotes platelet function in an autocrine manner by activating the S1PR_1_ receptor. However, as platelet activation reaches a level where thrombus formation needs to be restricted, higher concentrations of S1P subsequently limit platelet function and control the response through S1PR_4_ and S1PR_5_ receptors. The level of S1P release and local concentration is therefore uniquely placed to act as a natural switch between positive and negative priming of platelet function. Future studies will need to confirm whether S1P is a key player in the modulation of thrombus formation.

In conclusion, this study identified S1P as both a positive and negative platelet primer and demonstrates the underlying mechanism by which S1P affects PAR1-mediated platelet function in a concentration-dependent biphasic manner.

## Materials and methods

### Materials

Protease activated receptor 1 (PAR-1)-activating peptide (PAR1-AP/SFLLRN-NH_2_) was from Bachem (Bubendorf, Switzerland). Crosslinked collagen-related peptide (CRP-XL) from University of Cambridge (Cambridge, UK). Sphingosine-1-phosphate, FTY720, SEW2871, CYM5520, CYM5541, CYM50260, A971432, Ex 26, TY52156, JTE-013, CYM50358 hydrochloride, PF543 hydrochloride, N, N-dimethylsphingosine (DMS) were from Tocris (Bristol, UK). The D-erythro-Dihydrosphingosine-1-phosphate (DHS1P) was from Sigma Aldrich (Poole, UK). FTY720-Phosphate was from Insight Biotechnology (Wembley, UK). Mouse anti-human PAC-1 conjugated to FITC and mouse anti-human CD62P conjugated to PE were from BD Biosciences (Oxford, UK). Fura-2 AM cell permeant calcium indicator and ActinGreen 488 ReadyProbes Reagent were from ThermoFisher Scientific (Loughborough, UK). Unless indicated, all other materials were from Sigma Aldrich (Poole, UK).

### Isolation and preparation of human platelets

Venous blood was obtained from healthy volunteers in accordance with the Declaration of Helsinki and in agreement with the approval of the local ethic committee guidelines (University of Bristol). Donors provided written informed consent and confirmed they had not taken any antiplatelet drugs 14 days prior to donation. Blood was drawn into 4% trisodium citrate (1:9, v/v) and acidified with acid citrate dextrose (1:7, v/v). Washed human platelets were prepared as previously described^54^. Platelets were resuspended with modified HEPES Tyrode buffer (145 mM NaCl, 3 mM KCl, 0.5 mM Na_2_HPO_4_, 1 mM MgSO_4_.7H_2_O, 10 mM HEPES, pH 7.2, 0.1% D-glucose (w/v) and 0.02 U mL^−1^ apyrase) to a final density of 4 × 10^8^ mL^-1^ and rest for 30 min at 30 °C before experimentation.

### Platelet aggregation

Platelet aggregation was performed on a Chrono-log model 490 aggregometer (Labmedics, Oxfordshire, UK) under 1200 rpm stirring at 37 °C. Rested platelets (2 × 10^8^ mL^-1^) were pre-incubated with vehicle (0.2% methanol), S1P receptor agonists or antagonists for 5 min in prior to stimulation with PAR1 agonist (SFLLRN) or CRP at indicated concentrations. The changes in light transmission were measured and recorded on an AGGROLINK Version 4 software (Chronolog Corporation, Pennsylvania, USA) for 5 min.

### Platelet function assays

#### Flow cytometry

Human platelet integrin α_IIb_β_3_ activation and α-granule secretion were measured by flow cytometry on a BD Accuri™ C6 plus (BD Biosciences, Oxford, UK) flow cytometer. The FITC-conjugated antibody, which binds the activated integrin α_IIb_β_3_ and PE-conjugated antibody binds the α-granule marker CD62P were used. Platelets (2 × 10^7^ mL^-1^) were stimulated as indicated and fixed using 2% paraformaldehyde, then read on the flow cytometer until a total of 10,000 events were collected from each well.

#### Calcium mobilisation

Fura-2 AM calcium indicator was added to platelet rich plasma (1:1000 dilution, working concentration 4 μM for 60 min) before making washed platelets. Rested platelets (2 × 10^8^ mL^-1^) were supplemented with 2 mM calcium, then incubated with vehicle or S1P (100 nM or 10 μM) for 5 min at 37 °C. Platelet samples were loaded into 96 well plates and read on a Tecan Infinite M200 multimode plate reader (Mannedorf, Switzerland) and allowed 10 cycles to establish baseline. Fluorescence of fura-2 was measured as the ratio at wavelengths 340:380 nm. PAR1-AP was then added and responses were recorded from the next 40 cycles. For the last 10 cycles, Triton™ X-100, Ethylenediaminetetraacetic acid (EDTA) and Tris were added for calibration.

#### Human platelet spreading

Glass coverslips (13 mm) were placed into a 24-well plate and coated with fibrinogen (100 μg/mL) dissolved in HEPES-Tyrode’s for 2 h at room temperature. Subsequently the uncoated area of the well was blocked using 2% fatty-acid free BSA. Human platelets (2 × 10^7^ mL) were stimulated as indicated, then incubated in the wells for 60 min at 30 °C to allow cell spreading and adhesion. Platelets were fixed using 4% paraformaldehyde with non-adhered cells removed by PBS washes and permeabilised by 0.1% Triton™ X-100 to allow fluorescent stain entry. Platelets were loaded with ActinGreen 488 stain for 30 min. The coverslips were mounted using Prolong Diamond Antifade Mountant (Thermofisher Scientific) and set in a dark room overnight. The adhered platelets were imaged using Leica DMI6000 B fluorescent microscope with a 63 × A/1.4 oil objective (Leica Camera AG, Germany). The number of platelets adhered and surface area covered per 1000 μm^2^ was determined from three randomly selected fields of view per treatment condition using ImageJ software. The percentage of platelets in each phase (phase I = compact shape, phase II = Filopodial extension, phase III = filopodial extension and spread, phase IV = fully spread^55^) was assessed by counting the platelets in each phase and dividing by the total number of platelets. An average from three replicates per sample was calculated.

#### Data analysis

Data were analysed using GraphPad Prism 7 software (GraphPad Software, California, USA). All data are presented as the mean ± standard error of the mean with at least three independent experiments. Statistical tests used were as indicated in the figure legends, using a parametric test where data was normal and a non-parametric test where data did not show a normal distribution. We consider a *P* value of < 0.05 as significant. Concentration–response curves were plotted with a four-parameter logistic equation.

## Supplementary Information

Supplementary Figure 1.

Supplementary Figure 2.

Supplementary Figure Legends.

## Data Availability

The datasets generated during and/or analysed during the current study are available from the corresponding author on reasonable request.
