# Supplementary figures and images for: Sphingosine-1-phosphate modulates PAR1-mediated human platelet activation in a concentration-dependent biphasic manner
Source: Sci Rep. 2021 Jul 28;11:15308. doi: 10.1038/s41598-021-94052-4 (PMC8319165; doi:10.1038/s41598-021-94052-4)

# Supplementary Figure 1

**a (i)**

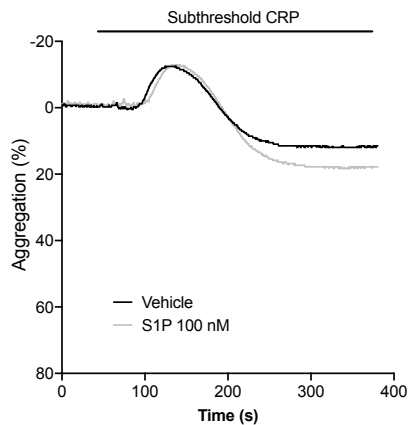

**(ii)**

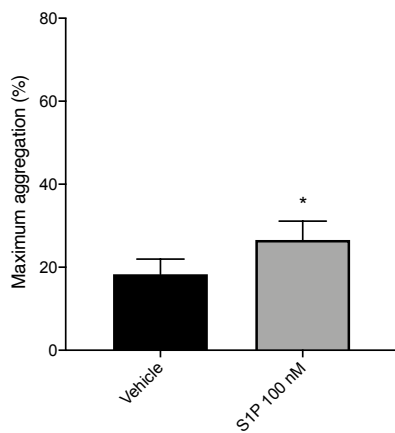

**b (i)**

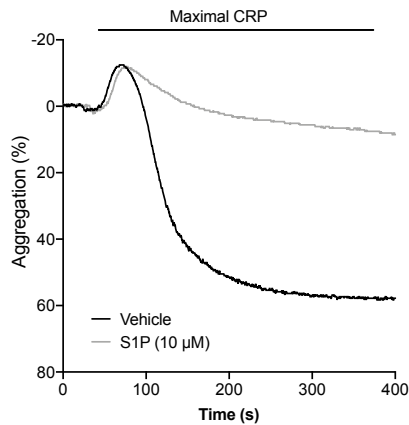

**(ii)**

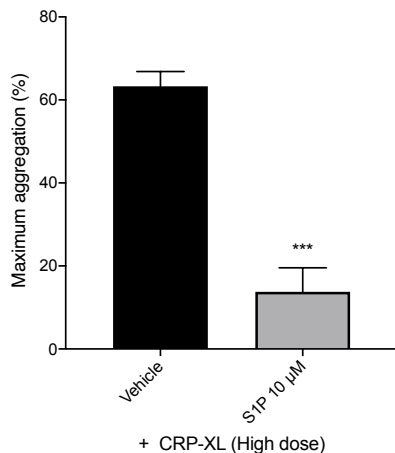

Supplement: Supplementary file 1 — Supplementary Figure 1. [file 41598_2021_94052_MOESM1_ESM.pdf]

# Supplementary Figure 2

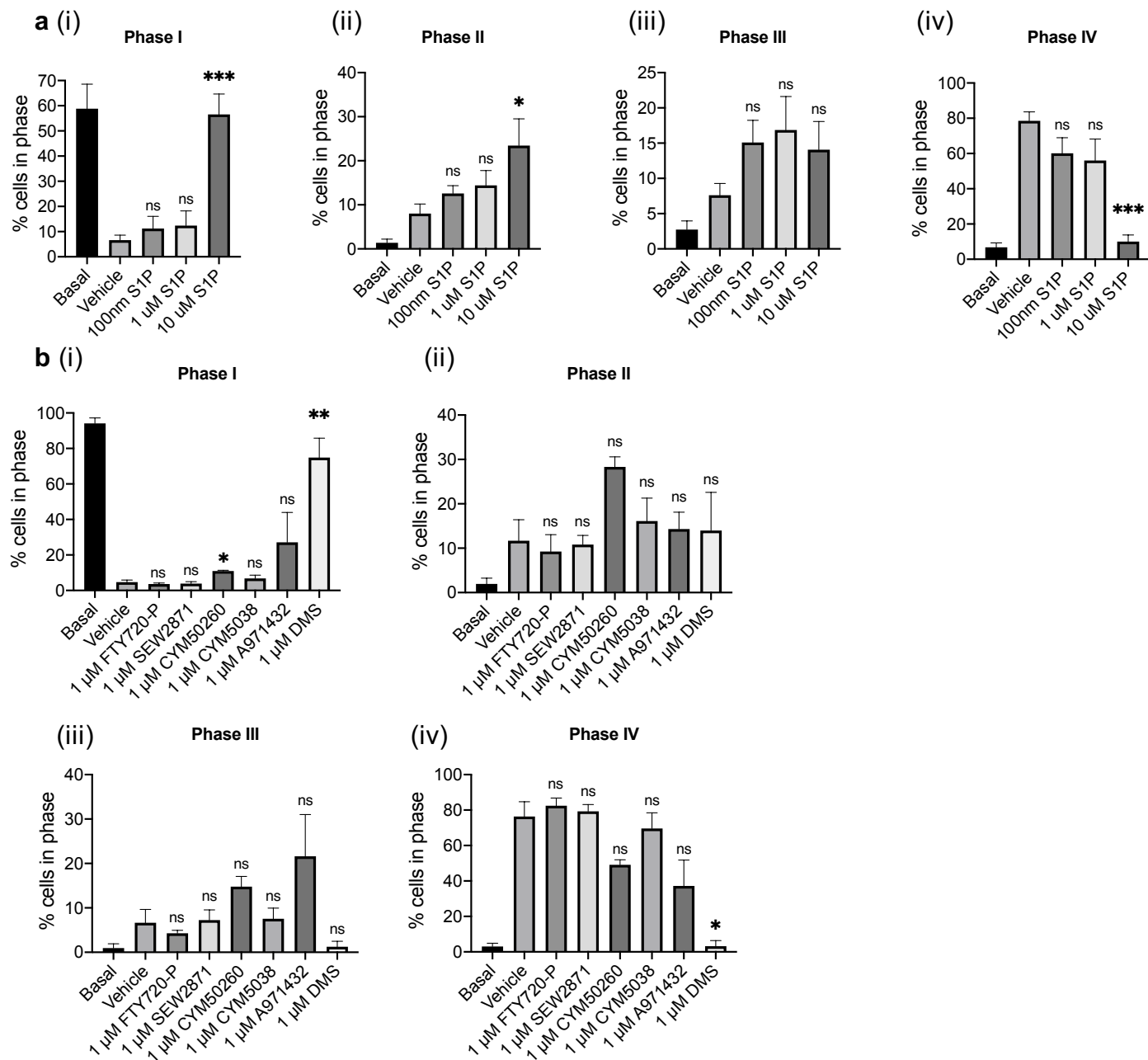

Supplement: Supplementary file 2 — Supplementary Figure 2. [file 41598_2021_94052_MOESM2_ESM.pdf]
