# Supplementary material for: Sphingosine-1-phosphate modulates PAR1-mediated human platelet activation in a concentration-dependent biphasic manner
Source: Sci Rep. 2021 Jul 28;11:15308. doi: 10.1038/s41598-021-94052-4 (PMC8319165; doi:10.1038/s41598-021-94052-4)
Supplement: Supplementary file 3 — Supplementary Figure Legends. [file 41598_2021_94052_MOESM3_ESM.docx]

**Supplementary Figure Legends**

**Supplementary Figure 1. S1P modulates CRP-induced platelet aggregation**

Washed human platelets (2 × 10^8^/mL) were pre-incubated with vehicle (0.2% methanol), 100 nM S1P and 10 S1P μM for 5 minutes. Platelet aggregation was induced by subthreshold or maximal concentrations of CRP and recorded for 5 minutes. (a) (i) Representative trace of subthreshold aggregation induced by CRP in the absence or presence of 100 nM S1P. (ii) Bar graph of quantified percentage maximum aggregation induced by subthreshold CRP alone or with the presence of 100nM S1P (Mean + SEM, N=3, student’s paired t-test, * P < 0.05). (b) (i) Representative trace of maximal aggregation induced by maximal CRP concentration in the absence or presence of 10 μM S1P (Mean + SEM, N=4, student’s paired t-test, *** P < 0.001).

**Supplementary Figure 2. Modulation of platelet phases by S1P, S1PR agonists, antagonists and SphK inhibitors from spreading assays**

Washed human platelets (7 × 10^7^/mL) were incubated with vehicle control (0.2% DMSO), concentrations of S1P, S1P receptor subtype specific agonists or SPHK inhibitor DMS for 10 minutes, samples were spread and adhered onto a 96 wells glass bottom plate coated with 100 μg/mL fibrinogen for 60 minutes at 37°C. Platelets were fixed for 10 minutes using 4% formaldehyde and permeabilised with 0.1% triton in prior to 30 minutes staining by ActinGreen 488 ReadyProbes reagent. Phase I = compact shape, phase II = filopodial extension, phase III = filopodial extension and spread, phase IV = fully spread. N=5 for following experiments, mean + SEM shown. Results analysed using a repeated measures one-way ANOVA with Dunnett’s multiple comparisons. P values shown for comparisons with vehicle response. ns = P > 0.05, * P <0.05, ** P < 0.01, *** P < 0.001 (a) Phases of platelet activation in response to 100 nM, 1 μM and 10 μM S1P (i) Phase I, (ii) Phase II, (iii) Phase III, (iv) Phase IV. (b) Phases of platelet activation in response to 1 μM FTY720 phosphate, 1 μM SEW2871, 1 μM CYM50260, 1 μM CYM50358 hydrochloride, 1 μM A971432, 1 μM DMS (i) Phase I, (ii) Phase II, (iii) Phase III, (iv) Phase IV.
